# Prevalence of pre-operative anxiety and associated factors among a group of women undergoing gynaecological surgeries at a single unit in a tertiary care hospital in Sri Lanka

**DOI:** 10.12688/f1000research.26964.1

**Published:** 2021-02-05

**Authors:** Madura Jayawardane, Wedisha Gankanda, Malsha Gunathilake

**Affiliations:** 1 Senior Lecturer, Department of Gynaecology and Obstetrics, Faculty of Medical Sciences, University of Sri Jayewardenepura, Nugegoda, Sri Lanka; 2 Acting Consultant in Gynaecology and Obstetrics, Colombo South Teaching Hospital, Kalubowila, Sri Lanka; 3 Senior Registrar in Psychiatry, National Institute of Mental Health, Angoda, Sri Lanka

**Keywords:** Pre-operative anxiety, Surgery related anxiety, Anxiety

## Abstract

**Background: **Surgery-related anxiety is universal, leading to complications. The objective of this study was to assess the prevalence of pre-operative anxiety levels among a group of patients.

**Methods: **A descriptive cross-sectional study of 64 women was conducted in a tertiary care hospital, Sri Lanka. Patients who underwent emergency surgeries, those with mental illnesses or those aged <18 years were excluded. Pre-operative assessment was done one day prior to the surgery using a self-administered Sinhala validated Amsterdam-Preoperative-Anxiety-and-Information-Scale (APAIS), Hospital Anxiety and Depression Scale (HADS) and Visual-Analogue-Scale (VAS). The APAIS consists of six questions which assess three anxiety components: anesthesia-related-anxiety (Sum A), surgery-related-anxiety (Sum S) and information-desire-component (Sum IDC). The combined score (Sum C) is given by the total of Sum A and Sum S. A Sum C of ≥11 indicates significant anxiety.

**Results: **The mean age of participants was 38.03 years (SD=13.53 years). The mean total score of APAIS was 10.36 (4.06), of HADS was 5.734 (4.487) and of VAS was 3.156 (2.773). All scores were higher in participants <50 years. There were negative correlations between age and anxiety levels in VAS and APAIS scales; the Sum IDC in ****APAIS (r=-0.416, p=0.001) and VAS scores (r=-246, p=0.050) showed significant negative correlations. Excepting Sum IDC all APAIS, HADS and VAS scores were higher among the group without insurance; despite free healthcare. However only Sum S (t=-3.716, p=0.000) and Sum C (t=-2.281, p=0.026) in APAIS, HADS (t=-3.412, p=0.001) and VAS (t=-2.135, p=0.037) had statistically higher values. Anxiety scores were higher in the group that underwent minor surgeries but where not significantly related to education level, marital status, income, employment or living status.

**Conclusions: **Pre-operative anxiety is common. Age <50 years, lacking insurance cover and undergoing minor surgeries are associated with increased pre-operative anxiety levels. Screening and appropriate interventions would be beneficial.

## Introduction

Anxiety is an emotional state described as a vague, uneasy feeling, the source of which is often nonspecific. It is known to cause abnormal hemodynamics as a consequence of sympathetic, parasympathetic and endocrine stimulation ^1^. Barlow described anxiety as a unique and coherent cognitive-affective structure within our defensive and motivational system ^2^. At the base there is a sense of uncontrollability focused largely on possible future threats, danger, or other upcoming potentially negative events ^2^. Anxiety can only be understood by taking into account some of its cognitive aspects, particularly because a basic aspect of anxiety appears to be uncertainty ^3^. The uncertainty regarding these situations highlights a lack of control that contributes to feelings of anxiety and makes coping more difficult ^4^. 

Patients awaiting surgery are more likely to develop anxiety in the pre-operative period. The study of qualitative aspects of anxiety reveals three distinct dimensions of preoperative fear: fear of the unknown, fear of feeling ill and fear for one’s life ^5^. Pre-operative anxiety has a substantial negative impact on the overall outcome of surgeries. Among them, post-operative pain is the commonest complication ^6^. Pre-operative anxiety has also been found to contribute to other post-operative problems such as nausea, vomiting, tachycardia, hypertension, and an increase in the risk of infection. Pre-operative anxiety in young children undergoing surgery is associated with a more painful postoperative recovery and a higher incidence of sleep related and other problems.

Studies have shown that a large proportion of surgical patients experience considerable levels of pre-operative anxiety. This figure stands at around 60%–80% in the Western population ^7, 8^. A study conducted in Ethiopia has shown that 70.3% of patients have had anxiety during the pre-operative period ^7, 9^. A study done among a group of patients awaiting general surgeries in Sri Lanka showed that the prevalence of anxiety was 76.7% ^1^.

There are certain identified factors which contribute to higher levels of pre-operative anxiety among patients, including female gender, younger age and absence of past surgical experience. The study conducted in Sri Lanka showed that females and those who have never undergone surgeries were more anxious than males and those who had previously undergone surgeries, respectively ^1^.

As women are identified as a vulnerable group, assessing the prevalence and factors that contribute to pre-operative anxiety among them is important as a means of identifying high risk individuals, and to plan interventions that minimize anxiety and related complications. 

The objective of this study was to assess the prevalence of pre-operative anxiety levels and related factors among a group of women awaiting elective gynaecological surgeries.

## Methods

### Study participants

A descriptive cross-sectional study was conducted from 1/July/2018 to 1/December/2019 at the Professorial Gynaecology Unit, Ward 23, Colombo South Teaching Hospital. A total of 64 patients who were 18 and over years of age who underwent major elective gynaecological surgeries were included in the study. Patients who were admitted to the ward were checked for eligibility and once consented they were recruited into the study. Patients who underwent emergency surgeries, patients with mental illnesses or those aged below 18 years were excluded from the study sample. The sample size was determined using a non-probability sampling method where all subjects who fulfilled the criteria during the study period were enrolled into the study. Biasness of the study was controlled by excluding subjects who had previously undergone surgeries, those who had previously been exposed to the theatre environment and subjects with psychiatric disorders.

### Data collection

The data was collected by an independent research person who did not have any directly involvement in the patients’ care during their period of hospital admission for the surgery. Pre-operative assessment was done on the day prior to the surgery. The anxiety level was assessed using the Sinhala version of the self-administered Amsterdam Preoperative Anxiety and Information Scale (APAIS) ^10^, Hospital Anxiety and Depression Scale (HADS) ^11^ and Visual Analogue Scale (VAS) ^8^. A pre-tested self-administered questionnaire was used to gather basic demographic data of the participants. All materials are provided as extended data ^12^.

The APAIS ^10^ is a self-administered questionnaire that is widely used to assess pre-operative anxiety. It consists of six questions which assess three components of anxiety; anesthesia-related anxiety (Sum A), surgery-related anxiety (Sum S) and information desire component (Sum IDC). Combined score (Sum C) is given by the total Sum of A and Sum S. Its Sinhala version has been validated in Sri Lanka ^1^. In the Sinhala version, a Sum C of 11 or more indicates a significantly anxious patient. VAS is used to measure levels of anxiety. It is a 10 cm size graduated line in which the left end is marked as 0 (“no anxiety”), and the right end is taken as 10 ("maximum anxiety”). The patients were advised to evaluate their own anxiety level and then mark it on the given line. The Hospital Anxiety and Depression Scale (HADS) ^11^ is also a self-administered scale that consists of 7 questions. The total is scored out of 21. A score of 0–7 is considered as normal, 8–10 and 11–21 are considered as borderline and abnormal anxiety levels respectively.

### Statistical analysis

SPSS version 17 was used to analyze the data. Pearson's correlation analysis was used to assess the correlation between each tool. A paired t-test was used to compare the mean scores of anxiety in different scales with the socio-demographic and surgical factors (age, education level, and marital status, availability of insurance coverage, living status, income and type of surgery) for the two randomized samples. A p value < 0.05 was taken as significant.

### Ethical consideration

Ethical approval was obtained from the Ethics Review Committee of the Faculty of Medical Sciences, University of Sri Jayewardenepura. Informed written consent was given by each participant. Identity and privacy of the data of the participants were handled with confidentiality accordingly.

## Results

A total of 64 women were included in the study ^13^. The mean age of the participants was 38.03 years (SD= 13.53 years, range- 20–83 years). The majority of them (56.3%) were awaiting major surgeries. Table 1 shows the basic demographic characteristics of the participants and the type of surgery they underwent.

**Table 1.** Basic demographic characteristics of the participants and the type of surgery they underwent.

Variable		Number (%)
Marital status	Married	55 (85.9)
	Single	7(10.9)
	Divorced	2 (3)
Level of education	Grade 1-5	5 (7.8)
	Grade 6-10	12 (18.8)
	A/L	35 (54.7)
	Higher education	12 (18.8)
Occupation	Employed	32 (50)
	Un-employed	32 (50)
Monthly family income	<10000	3 (4.5)
	10000- 25000	9 (14.1)
	25000-50000	34 (53.1)
	50000-100000	13 (20.3)
	>100 000	5 (7.8)
Having a health insurance	Yes	19 (29.7)
	No	45 (70.2)
Living	With family	57 (89.1)
	Alone	7 (10.9)
Type of surgery	Major	36 (56.3)
	Minor	28(43.8)

### Pre-operative anxiety level scored in the three scales

Table 2 summarizes the mean scores of three scales. According to the Sum C values in APAIS, 26 (40.6%) had significant pre-operative anxiety levels. According to the total score in HADS, 11 (17.2%), 6 (9.4%) and 47 (73.4%) had abnormal, borderline and normal levels of anxiety, respectively.

**Table 2.** Mean scores of Amsterdam Preoperative Anxiety and Information Scale, Hospital Anxiety and Depression Scale and Visual Analogue Scale.

	Components	Mean (SD)
Amsterdam Preoperative Anxiety and Information Scale	Anaesthesia related anxiety (Sum A)	4.67 (2.30)
	Surgery related anxiety (Sum S)	6.28 (2.40)
	Information Desire component (Sum IDC)	6.16 (2.72)
	Combined Anxiety component (Sum C)	10.36 (4.06)
Hospital Anxiety and Depression Scale		5.734 (4.49)
Visual Analogue Scale		3.156 (2.77)

The correlation between information desire component (Sum IDC) and the total score (Sum C) was assessed using the Pearson's correlation. There was no significant correlation between the two scores (r=0.094, p=0.462).

### Comparison of three scales

 Pearson's correlation was used to assess the correlations between Sum C in APAIS, HADS and VAS. All three showed significant correlation with each other; Sum C and HADS (r=0.481, p=0.000), Sum C and VAS (r=0.470, p=0.000), HAD and VAS (r=0.678, p= 0.000).

All the scores were higher in the <50 years of age category than in the older group ( Table 3). Only the Sum IDC had a significant difference (t=2.875, P= 0.006). There were negative correlations between age and anxiety levels in the VAS and APAIS scales. But out of them, Sum IDC in APAIS (r=-0.416, p=0.001) and VAS scores (r=-246, p=0.050) showed significant negative correlations.

**Table 3.** Factors associated with anxiety. The data is represented as mean values.

Factor		Sum A (out of 10)	Sum S (out of 10)	Sum IDC (out of 10)	Sum C (out of 20)	HADS (out of 21)	VAS (out of 10)
Age	<50	4.611	6.426	6.556 *	10.740	5.703	3.333
	≥50	3.700	5.500	4.000 *	8.300	5.900	2.200
Level of education	Up to O/L	4.118	6.235	5.294	9.353	6.000	2.470
	Above O/L	4.596	6.298	6.468	10.723	5.638	3.404
Marital status	Married	4.545	6.309	6.218	10.564	6.000	3.345
	Single/Divorced/Widow	4.000	6.111	5.778	9.111	4.111	2.000
Having a Health insurance	Yes	4.000	4.632 *	6.421	8.631 *	2.947 *	2.000 *
	No	4.750	6.955 *	5.977	11.114 *	6.818 *	3.568 *
Monthly income	< 50, 000	4.844	6.089	6.311	10.356	5.511	3.133
	≥ 50,000	3.666	6.889	5.889	10.611	6.555	3.388
Employment	Employed	4.406	6.281	6.625	10.218	4.906	3.156
	Unemployed	4.531	6.281	5.687	10.500	6.562	3.156
Living status	With family	4.280	6.175	6.210	10.158	5.649	3.193
	Alone	6.000	7.143	5.714	12.000	6.428	2.857
Type of surgery	Minor	5.178 *	6.571	7.750 *	11.178	6.107	4.107 *
	Major	3.916 *	6.056	4.917 *	9.722	5.444	2.416 *

*p<0.05 (significantly different mean values).

Except Sum IDC, all scores in APAIS, HADS and VAS were higher among the group without an insurance. Out of the sub-scores, Sum S (t=-3.716, p=0.000) and Sum C (t=-2.281, p=0.026) in APAIS, HADS (t=-3.412, p=0.001) and VAS (t=-2.135, p=0.037) had statistically higher values.

All the anxiety scores were higher in the group that underwent minor surgeries. Out of all, Sum A (t=2.237, p=0.029), Sum IDC (t=4.788, p=<0.001) scores in APAIS and VAS (t=2.520, p= 0.014) showed significantly higher values.

Education level, marital status, income, employment and living status did not show significant associations with anxiety levels (p>0.05).

## Discussion

The prevalence of pre-operative anxiety in western studies varies from 30–80% ^1^. In this study, the prevalence of anxiety was 40.6%. However, a study performed among Sri Lankan general surgical patients using the same APAIS score and cutoff values has found a higher prevalence of 76.7% of pre-operative anxiety ^1^. They also found that patients with higher information desire (Sum IDC) had more anxiety, whereas in the present study there was no significant correlation between the information desire and anxiety.

There were significant positive correlations among all three scales. Past studies, including the Sri Lankan study ^1, 14– 16^, have identified various socio-demographic and surgery related factors contributing to pre-operative anxiety. In the Sri Lankan study the age, education, marital status, category of surgery (major/minor), income and having an insurance coverage were not associated with pre-operative anxiety but this study showed pre-operative anxiety scores of APAIS and VAS were negatively correlated with age ^1^. Out of them, VAS score and the Sum IDC score of APAIS had significant negative correlations with age. Therefore, it is likely that the information desire and levels of pre-operative anxiety reduce with ageing. Furthermore, it was also identified that not having insurance had significantly higher scores in all three scales this is despite the healthcare being free in the study setting. Paradoxically, patients who were undergoing minor surgeries had significantly higher Information desire (Sum IDC) scores and VAS scores compared to those undergoing major surgeries. These findings were contradicting to the findings of former studies ^1^. This may be due to less comprehensive informed consenting practiced for minor surgeries compared to major surgeries in the study setting. There were findings similar to previous studies such no significant associations between levels of pre-operative anxiety and education level, marital state, living alone or with family, employment status and income.

As this study is part of a clinical trial, prior experience in undergoing surgery or anaesthesia was an exclusion criterion. Therefore, the effect of prior experience on pre-operative anxiety was not assessed.

## Conclusions

Overall, 40.6% of patients undergoing elective gynecological surgeries suffer from anxiety. Age less than 50 years, not having an insurance coverage and undergoing minor surgeries increases the pre-operative anxiety levels in this group. Therefore, screening, early identification and appropriate interventions for those with the above risk factors are may be beneficial to reduce pre-operative anxiety levels.

## Data availability

### Underlying data

Harvard Dataverse: Prevalence of pre-operative anxiety and associated factors among a group of women undergoing gynaecological surgeries at a single unit in a tertiary care hospital in Sri Lanka. https://doi.org/10.7910/DVN/KN3AUL ^13^.

The project contains the following underlying data:

- Prevelance of Pre-operative.tab (Dataset)

### Extended data

Harvard Dataverse: Prevalence of pre-operative anxiety and associated factors among a group of women undergoing gynaecological surgeries at a single unit in a tertiary care hospital in Sri Lanka. https://doi.org/10.7910/DVN/4QMH3Z ^12^.

The project contains the following extended data:

- F1000_Prevalence of Pre-op_Extended data.docx (Data collection sheets)

Data are available under the terms of the Creative Commons Zero "No rights reserved" data waiver (CC0 1.0 Public domain dedication).

## References

[1] Matthias AT Samarasekera DN: Preoperative anxiety in surgical patients - experience of a single unit. *Acta Anaesthesiol Taiwan.* 2012;50(1):3–6. 10.1016/j.aat.2012.02.004 22500906

[2] Barlow DH: Unraveling the mysteries of anxiety and its disorders from the perspective of emotion theory. *Am Psychol.* 2000;55(11):1247–63. 10.1037//0003-066x.55.11.1247 11280938

[3] Strongman KT: The Psychology of Emotion: Theories of Emotion in Perspective. In: *Behavioural and Cognitive Psychotherapy*. Chichester, UK: Cambridge University Press, Wiley;1997;383.

[4] Craig KJ Brown KJBA: Environmental factors in the etiology of anxiety. Psychopharmacology: the Fourth Generation of Progress. In: *Environmental factors in the etiology of anxiety Psychopharmacology: the Fourth Generation of Progress*. New York, NY: Raven Press;1995;1325–1339.

[5] Kain ZN Mayes LC Caldwell-Andrews AA: Preoperative anxiety, postoperative pain, and behavioral recovery in young children undergoing surgery. *Pediatrics.* 2006 [cited 2020 Aug 19];118(2):651–8. 10.1542/peds.2005-2920 16882820

[6] Trumper M: Bodily Changes in Pain, Hunger, Fear and Rage: An Account of Recent Researches into the Function of Emotional Excitement. *Psychol Clin.* 1930 [cited 2020 Aug 19];19(3):100–101.

[7] Nigussie S Belachew T Wolancho W: Predictors of preoperative anxiety among surgical patients in Jimma University Specialized Teaching Hospital, South Western Ethiopia. *BMC Surg.* 2014 [cited 2020 Aug 19];14(1):67. 10.1186/1471-2482-14-67 25189274 PMC4167307

[8] Kindler CH Harms C Amsler F: The visual analog scale allows effective measurement of preoperative anxiety and detection of patients' anesthetic concerns. *Anesth Analg.* 2000 [cited 2020 Aug 19];90(3):706–12. 10.1097/00000539-200003000-00036 10702461

[9] Bedaso A Ayalew M: Preoperative anxiety among adult patients undergoing elective surgery: a prospective survey at a general hospital in Ethiopia. *Patient Saf Surg.* 2019 [cited 2020 Aug 19];13(1):18. 10.1186/s13037-019-0198-0 31007718 PMC6454677

[10] Celik F Edipoglu IS: Evaluation of preoperative anxiety and fear of anesthesia using APAIS score. *Eur J Med Res.* 2018 [cited 2020 Aug 19];23(1):41. 10.1186/s40001-018-0339-4 30205837 PMC6131845

[11] Stern AF: Questionnaire review the Hospital anxiety and Depression scale. *Occup Med (Chic Ill).* 2014 [cited 2020 Aug 19];64(1):393–4. Reference Source 10.1093/occmed/kqu024 25005549

[12] Jayawardane M Gankanda WI Gunathilake IAGMP: Prevalence of pre-operative anxiety and associated factors among a group of women undergoing gynaecological surgeries at a single unit in a tertiary care hospital in Sri Lanka. Harvard Dataverse. Harvard Dataverse, V1.2021. 10.7910/DVN/4QMH3Z PMC8127014 34055313

[13] Jayawardane M Gankanda WI Gunathilake IAGMP: Prevalence of pre-operative anxiety and associated factors among a group of women undergoing gynaecological surgeries at a single unit in a tertiary care hospital in Sri Lanka. Harvard Dataverse. 2021. Harvard Dataverse, V1, UNF:6:BaYrbsHy8UMdA2D69dFkkQ== [fileUNF];2021. 10.7910/DVN/KN3AUL PMC8127014 34055313

[14] Moerman N van Dam FS Muller MJ: The Amsterdam Preoperative Anxiety and Information Scale (APAIS). *Anesth Analg.* 1996 [cited 2020];82(3):445–51. 10.1097/00000539-199603000-00002 8623940

[15] Nishimori M Moerman S Fukuhara S: Translation and validation of the Amsterdam preoperative anxiety and information scale (APAIS) for use in Japan. *Qual Life Res.* 2002 [cited 2020 Aug 19];11(4):361–64. 10.1023/a:1015561129899 12086121

[16] Apinya K Siriporn P Puchong L: Validity and reliability of the Amsterdam Preoperative anxiety and Information Scale (APAIS); Thai version in adult Thai pre-operative patients. *Journal of Psychiatric Association of Thailand.* 2009 [cited 2020 Aug 19];54:86–9. Reference Source

