# Supplementary material for: Prevalence of pre-operative anxiety and associated factors among a group of women undergoing gynaecological surgeries at a single unit in a tertiary care hospital in Sri Lanka
Source: F1000Res. 2021 Feb 5;10:74. [Version 1] doi: 10.12688/f1000research.26964.1 (PMC8127014; doi:10.12688/f1000research.26964.1)
Supplement: Peer review history

# Peer review: Reviewer response for version 1

**DOI:** 10.5256/f1000research.29785.r82932

**Published:** 2021-05-12

**Authors:** Thilina Jayarathne

**Affiliations:** 1 Purdue University, West Lafayette, IN, USA

This is a sound cross-sectional study. The scales used in this study are validated in the same setup and internationally. Pre-operative anxiety is a source of negative surgical outcome like prolonged recovery, nausea, vomiting, increased analgesia needs and post-operative infection, therefore this study fills several scientific gaps regarding the same. This study concludes pre-operative anxiety is common in a cohort of gynecological surgical patients in Sri Lanka whereas a similar study has proven higher prevalence in a cohort of general surgical patients appreciating the differences between specialties. The prevalence is also lower compared to worldwide data; further proving the value of studying regional differences. Paradoxical higher information desire in patients undergoing minor surgeries compared to major surgeries is an important finding which could be intervened. The results are well presented in tabular form, well discussed comparing with local and international studies.

 Overall, this study could be concluded as an impressive scientific study and I recommend it to be indexed without major alterations.

Is the work clearly and accurately presented and does it cite the current literature?

Yes

If applicable, is the statistical analysis and its interpretation appropriate?

Yes

Are all the source data underlying the results available to ensure full reproducibility?

Yes

Is the study design appropriate and is the work technically sound?

Yes

Are the conclusions drawn adequately supported by the results?

Yes

Are sufficient details of methods and analysis provided to allow replication by others?

Yes

Reviewer Expertise:

Biologics

I confirm that I have read this submission and believe that I have an appropriate level of expertise to confirm that it is of an acceptable scientific standard.

# Peer review: Reviewer response for version 1

**DOI:** 10.5256/f1000research.29785.r79008

**Published:** 2021-03-22

**Authors:** Henna Rather

**Affiliations:** 1 Women's Division, Whipps Cross Hospital, Bart's Health NHS Trust, London, UK

- A sound cross-sectional study.
- Clearer description in tabular format of inclusion and exclusion criteria and how these were derived would be beneficial. 
- There are a few grammatical errors which need to be corrected.
- It would be beneficial to provide the sample questionnaires/scales (possibly as an appendix) that were given to the patient to assess anxiety levels.
- An expansion on results and discussion would be advantageous. It would be beneficial to expand the results obtained of other factors affecting anxiety listed in table 3, even if not statistically significant, particularly the link between pre-operative anxiety and information desire.
- It would also make the article more comprehensive if, in the discussion, some comparison is made to conclusions found in other studies on pre-operative anxiety around the world.

Is the work clearly and accurately presented and does it cite the current literature?

Partly

If applicable, is the statistical analysis and its interpretation appropriate?

Yes

Are all the source data underlying the results available to ensure full reproducibility?

Yes

Is the study design appropriate and is the work technically sound?

Yes

Are the conclusions drawn adequately supported by the results?

Yes

Are sufficient details of methods and analysis provided to allow replication by others?

Yes

Reviewer Expertise:

Obstetric and gynaecological research.

I confirm that I have read this submission and believe that I have an appropriate level of expertise to confirm that it is of an acceptable scientific standard, however I have significant reservations, as outlined above.

# Peer review: Reviewer response for version 1

**DOI:** 10.5256/f1000research.29785.r79007

**Published:** 2021-02-23

**Authors:** Chathurie Uththarika Suraweera

**Affiliations:** 1 Department of Psychological Medicine, University of Colombo, Colombo, Sri Lanka

- A good study.
- The language needs to be corrected at places.
- Explain how the participants were checked for exclusion criteria. Justify excluding participants with mental illnesses. I suggest to re-visit and expand the results section. A higher level of interpretation of the results would have added more scientific rigour to the discussion.

Is the work clearly and accurately presented and does it cite the current literature?

Yes

If applicable, is the statistical analysis and its interpretation appropriate?

Yes

Are all the source data underlying the results available to ensure full reproducibility?

Yes

Is the study design appropriate and is the work technically sound?

Yes

Are the conclusions drawn adequately supported by the results?

Partly

Are sufficient details of methods and analysis provided to allow replication by others?

Yes

Reviewer Expertise:

Psychiatry

I confirm that I have read this submission and believe that I have an appropriate level of expertise to confirm that it is of an acceptable scientific standard, however I have significant reservations, as outlined above.
